# Prevalence and treatment of postobstructive pneumonia among older adults with advanced cancer

**DOI:** 10.1017/ash.2022.293

**Published:** 2022-09-08

**Authors:** Seohyuk Lee, Lisa O’Donovan, Avi J. Cohen, Samir Gautam, Vincent Quagliarello, Manisha Juthani-Mehta, Rupak Datta

**Affiliations:** 1 Department of Internal Medicine, Yale School of Medicine, New Haven, Connecticut; 2 Section of Pulmonary and Critical Care Medicine, Yale School of Medicine, New Haven, Connecticut; 3 Section of Infectious Diseases, Yale School of Medicine, New Haven, Connecticut; 4 Hospital Epidemiology and Infection Prevention Program, Veterans Affairs Connecticut Healthcare System, West Haven, Connecticut

## Abstract

Among 124 older adults with advanced cancer who were hospitalized with pneumonia, 7.3% met criteria for postobstructive pneumonia. There were no differences in antibiotic duration, antibiotic spectrum, 30-day and 90-day readmissions, or mortality between those with and without postobstructive pneumonia. Bacteria were identified in 5 patients with postobstructive pneumonia.

Postobstructive pneumonia is an important complication of cancer,^1^ but its prevalence in hospitalized patients remains unknown. One report suggested that 45%–55% of patients with lung cancer develop pneumonia with a postobstructive component.^2^ A second showed that 2% of inpatients with community-acquired pneumonia have postobstructive pathology.^2^ However, definitions of postobstructive pneumonia have varied, and its prevalence has not been examined among high-risk older adults with advanced cancer.

Data regarding the occurrence and management of postobstructive pneumonia in high-risk populations may inform recommendations by antibiotic stewardship programs because treatment regimens are often broad-spectrum and prolonged.^1,2^ We evaluated the prevalence and management of postobstructive pneumonia among older adults with advanced cancer who were hospitalized with pneumonia.

## Methods

We studied a cohort of patients aged ≥65 years with advanced cancer who were hospitalized with non–ventilator-associated pneumonia after receiving palliative chemotherapy between January 1, 2016, and September 30, 2017, at Yale New Haven Hospital, a 1,541-bed tertiary-care teaching center that includes the Smilow Cancer Hospital. Advanced cancer was defined as stage III–IV solid tumors, stage III–IV lymphomas, as well as acute, refractory, relapsed, or active liquid tumors requiring chemotherapy or targeted therapies. Advanced cancer was identified using *International Classification of Diseases, Tenth Revision* (ICD-10) codes and confirmed via pathology reports or medical record review. Cases of non–ventilator-associated pneumonia among hospitalized patients were restricted to the index event occurring after receipt of palliative chemotherapy and met standardized criteria for clinically defined pneumonia.^4^ We identified the subset of patients with definite postobstructive pneumonia, defined as a pulmonary infiltrate that occurred exclusively distal to an obstructed bronchus, or probable postobstructive pneumonia, defined as a pulmonary infiltrate that occurred contiguous with but not exclusively distal to an obstructed bronchus. Pulmonary infiltrates were assessed using chest computed tomography or chest radiographs when chest computed tomography data were not available. When findings from chest computed tomography and chest radiographs were discordant, results from the former were recorded. This study was approved by the Yale Human Investigation Committee (IRB protocol ID no. 2000021599).

For each patient, we collected information regarding patient-level and hospitalization-level characteristics. We evaluated inpatient microbiological test results including lower respiratory cultures, blood cultures, urine antigen tests, nasal swabs, and respiratory pathogen panels. Antibiotics administered orally, intravenously, or intramuscularly were recorded from pharmacy data and confirmed by medical record review. For antibiotics, we determined inpatient and postdischarge length of therapy and antibiotic spectrum index, a metric designed to compare spectrum of activity ranging from 1 (eg, dicloxacillin) to 13 (eg, tigecycline). The mean antibiotic spectrum index per patient per indication was recorded.^5,6^

For all patients, we determined all-cause readmissions and all-cause mortality within 30 days and 90 days of discharge. Among patients with postobstructive pneumonia who died, we further determined whether postobstructive pneumonia was associated with death. Descriptive characteristics were compared using the Fisher exact test. Differences in antibiotic spectrum index and length of therapy were assessed using the Mann-Whitney *U* test. All analyses were performed in R version 3.6.2 software (R Foundation for Statistical Computing, Vienna, Austria). *P* < .05 was considered statistically significant.

## Results

We identified 124 older adults with advanced cancer who were hospitalized with non–ventilator-associated pneumonia (Table 1), including 12 patients who developed non–ventilator-associated pneumonia after hospital day 3. Overall, 7.3% of patients met criteria for definite or probable postobstructive pneumonia. Common clinical features upon presentation included tachypnea (n = 9), worsening gas exchange (n = 8), and white blood-cell count abnormalities (n = 7); fever was uncommon (n = 2). Chronic heart disease was less common among patients with postobstructive pneumonia (55.6%) versus those without (93.9%) postobstructive pneumonia (*P* = .003). Lung cancer was more common in patients with postobstructive pneumonia than in those without postobstructive pneumonia (66.7% vs 27.0%; *P* = .02). Types of lung cancer in those with postobstructive pneumonia included squamous cell carcinoma (n = 3), adenocarcinoma (n = 2), and small-cell carcinoma (n = 1).

**Table 1.** Descriptive Characteristics of Study Cohort According to Presence of Postobstructive Pneumonia

Characteristic	Postobstructive Pneumonia		*P* Value
	Criteria Met (n=9), No. (%)	Criteria Not Met (n=115), No. (%)	
**Age**			
65–74 y	6 (66.7)	49 (42.6)	.18
75–84 y	3 (33.3)	51 (44.3)	.73
85+ y	0 (0)	15 (13.0)	.60
**Sex**			
Male	8 (88.9)	72 (62.6)	.16
Female	1 (11.1)	43 (37.4)	
**Race**			
White	8 (88.9)	97 (84.3)	1.00
Black	1 (11.1)	7 (6.1)	
Other	0 (0)	11 (9.6)	
**Ethnicity**			
Hispanic	0 (0)	6 (5.2)	1.00
Non-Hispanic	9 (100)	109 (94.8)	
**Cancer type**			
**Liquid tumor**			
Leukemia/Lymphoma	0 (0)	20 (17.4)	.35
Multiple Myeloma	1 (11.1)	14 (12.2)	1.00
**Solid tumor**			
Gastrointestinal	0 (0)	19 (16.5)	.35
Genitourinary	1 (11.1)	17 (14.8)	1.00
Lung	6 (66.7)	31 (27.0)	.02
Other	1 (11.1)	14 (12.2)	1.00
**Comorbidities^a^**			
Chronic heart disease	5 (55.6)	108 (93.9)	.003
Chronic renal disease	1 (11.1)	14 (12.2)	1.00
Chronic pulmonary disease	5 (55.6)	47 (40.9)	.49
Chronic liver disease	0 (0)	5 (4.3)	1.00
Neurologic disease	0 (0)	19 (16.5)	.35
**Prior multidrug-resistant organisms^a^**			
MRSA	1 (11.1)	10 (8.7)	.58
VRE	1 (11.1)	15 (13.0)	1.00
ESBL	0 (0)	2 (1.7)	1.00
CRE	0 (0)	3 (2.6)	1.00
**Hospital characteristics^b^**			
Intensive care unit admission	3 (33.3)	25 (21.7)	.42
Intubation	2 (22.2)	12 (10.4)	.27
Bronchoscopy	5 (55.6)	85 (73.9)	.26
Chest computed tomography	9 (100)	72 (62.6)	.03
**Length of stay**			
<7 d	2 (22.2)	57 (49.6)	.17
7–14 d	5 (55.6)	46 (40.0)	.49
>14 d	2 (22.2)	12 (10.4)	.27
**All-cause readmissions**			
30 d	4 (44.4)	29 (25.7)	.25
90 d	5 (55.6)	48 (41.7)	.50
**All-cause mortality^c^**			
30 d	2 (22.2)	27 (23.9)^7^	1.00
90 d	5 (55.6)	38 (33.6)^7^	.17

Note. MRSA, methicillin-resistant *Staphylococcus aureus;* VRE, vancomycin-resistant *Enterococcus*; ESBL, extended-spectrum β-lactamase–producing Enterobacterales; CRE, carbapenem-resistant Enterobacterales.

a Assessed in the year prior to admission.

b Some patients had >1 comorbidity or hospital characteristic.

c Survival status unknown, n=2.

Microbiological testing revealed an organism in 55.6% of patients with postobstructive pneumonia versus 37.4% of patients without postobstructive pneumonia. Organisms identified in those with postobstructive pneumonia included *Streptococcus pneumoniae* from a urine antigen test, *Bacillus cereus* and *Haemophilus influenzae* from blood cultures, and *Stenotrophomonas maltophilia* and *Escherichia coli* from lower respiratory cultures.

Antibiotic spectrum index per patient was no different for those with postobstructive pneumonia (median, 6.2; interquartile range [IQR], 6.0–6.3) versus those without postobstructive pneumonia (median, 6.0; IQR, 5.2–6.4; *P* = .32) (Table 2). Among those with postobstructive pneumonia, 5 patients underwent therapeutic thoracenteses, 2 underwent tumor debulking to relieve obstruction, and 4 received palliative care consultation. Most patients (55.6%) with postobstructive pneumonia and 67% of patients without postobstructive pneumonia received postdischarge antibiotics. Length of therapy was not different between patients with postobstructive pneumonia (median, 12 days; IQR, 9–20) versus those without postobstructive pneumonia (median, 11 days; IQR, 8–13.5; *P* = .25).

**Table 2.** Characteristics of Antibiotic Therapy Among Hospitalized Older Adults With Advanced Cancer According to Presence of Postobstructive Pneumonia

Characteristic	Postobstructive Pneumonia	
	Criteria Met (n=9), No. (%)	Criteria Not Met (n=115), No. (%)
**Antibiotic class^a^**		
Aminoglycoside	0 (0)	1 (0.9)
β-lactam/β-lactam inhibitor	9 (100)	67 (58.3)
Carbapenem	0 (0)	4 (3.5)
1st- or 2nd-generation cephalosporin	1 (11.1)	21 (18.3)
3rd- or 4th-generation cephalosporin	4 (44.4)	70 (60.9)
Fluoroquinolone	3 (33.3)	54 (47.0)
Glycopeptide	9 (100)	82 (71.3)
Lincosamide	0 (0)	3 (2.6)
Macrolide	0 (0)	18 (15.7)
Metronidazole	1 (11.1)	12 (10.4)
Penicillin	9 (100)	70 (60.9)
Sulfonamide	1 (11.1)	10 (8.7)
Tetracycline	3 (33.3)	52 (45.2)
Trimethoprim	0 (0)	10 (8.7)
**Length of therapy**		
<7 d	0 (0)	11 (9.6)
7–14 d	6 (66.7)	81 (70.4)
>14 d	3 (33.3)	23 (20.0)

a Some patients received >1 antibiotic class.

All-cause mortality and all-cause readmissions were similar between groups (Table 1). Among patients with postobstructive pneumonia who died, both deaths within 30 days and 80% of deaths within 90 days were associated with postobstructive pneumonia.

## Discussion

In this cohort of older adults with advanced cancer who were hospitalized with non–ventilator-associated pneumonia, 7.3% of patients were identified with postobstructive pneumonia. Consistent with prior studies, lung cancer was associated with the development of postobstructive pneumonia, microbiological evidence of bacterial infection was often lacking, and mortality was high among older adults with advanced cancer.^1–3,7^ However, we observed no differences in antibiotic duration, antibiotic spectrum index, all-cause readmissions, or all-cause mortality between those with and without postobstructive pneumonia. These findings may be attributable, at least in part, to institutional practices and a study cohort consisting of older adults with advanced cancer. Further investigation is needed to establish the optimal duration of therapy for postobstructive pneumonia in high-risk populations.

Previous studies were limited by broad definitions of postobstructive pneumonia or the inclusion of only those with pulmonary malignancies.^1,2^ In contrast, our cohort included a heterogeneous population from a comprehensive cancer center who were hospitalized with pneumonia. When applying standardized criteria, we showed that the prevalence of postobstructive pneumonia may be lower than previously reported.^1^ Additionally, differences in mortality between those with and without postobstructive pneumonia appeared attenuated among older adults with advanced cancer.^7^

Although we observed protracted courses of broad-spectrum therapy in our cohort, the role of bacteria in the pathogenesis of postobstructive pneumonia remains uncertain. Prior evidence has indicated that accumulating or retained epithelial secretions contribute to radiographic findings and cause an obstructive pneumonitis.^8^ Several studies have suggested that bacterial etiologies were rarely identified, and polymicrobial flora recovered from diagnostic tests often represented colonization.^2,7^ Given that clinical and radiographic features were used to define postobstructive pneumonia rather than microbiological data, it is possible that obstructive pneumonitis was present in some patients meeting criteria for postobstructive pneumonia.

Our study had several limitations. It was conducted in a single center, with small sample size. We relied on medical record review, which may be prone to observation bias. Additionally, we used both computed tomography and radiograph data, which may have introduced measurement bias. Nevertheless, we showed that postobstructive pneumonia may be uncommon when applying standardized criteria, and previously reported differences in antibiotic regimens and clinical outcomes may be attributable to patient case mix. Our work supports implementation guidelines to reduce antibiotic use in terminally ill patients and highlights an opportunity for collaboration between antibiotic stewardship programs and palliative care providers.^9,10^

## References

[1] Rolston KVI , Nesher L. Postobstructive pneumonia in patients with cancer: a review. Infect Dis Ther 2018;7:29–38. 29392577 10.1007/s40121-018-0185-2 PMC5840104

[2] Rolston KV. Postobstructive pneumonia in cancer patients. Clin Infect Dis 2016;63:707–708. 27318331 10.1093/cid/ciw368

[3] Marrie TJ. Pneumonia and carcinoma of the lung. J Infect 1994;29:45–52. 7963634 10.1016/s0163-4453(94)95060-1

[4] 2022 National Healthcare Safety Network Patient Safety Manual. Pneumonia (ventilator-associated [VAP] and non–ventilator-associated pneumonia [PNEU]) Event. Centers for Disease Control and Prevention website. https://www.cdc.gov/nhsn/pdfs/pscmanual/6pscvapcurrent.pdf. Published 2022. Accessed April 25, 2022.

[5] Leung V , Li M , Wu JH , et al. Evaluating antimicrobial use and spectrum of activity in Ontario hospitals: feasibility of a multicentered point-prevalence study. Open Forum Infect Dis 2018;5:ofy110. 29977965 10.1093/ofid/ofy110 PMC6016426

[6] Gerber JS , Hersh AL , Kronman MP , Newland JG , Ross RK , Metjian TA. Development and application of an antibiotic spectrum index for benchmarking antibiotic selection patterns across hospitals. Infect Control Hosp Epidemiol 2017;38:993–997. 28560946 10.1017/ice.2017.94

[7] Abers MS , Sandvall BP , Sampath R , et al. Postobstructive pneumonia: an underdescribed syndrome. Clin Infect Dis 2016;62:957–961. 26908806 10.1093/cid/civ1212 PMC4803103

[8] Burke M , Fraser R. Obstructive pneumonitis: a pathologic and pathogenetic reappraisal. Radiology 1988;166:699–704. 3340764 10.1148/radiology.166.3.3340764

[9] Barlam TF , Cosgrove SE , Abbo LM , et al. Implementing an antibiotic stewardship program: guidelines by the Infectious Diseases Society of America and the Society for Healthcare Epidemiology of America. Clin Infect Dis 2016;62:e51–e77. 27080992 10.1093/cid/ciw118 PMC5006285

[10] Datta R , Topal J , McManus D , et al. Perspectives on antimicrobial use at the end of life among antibiotic stewardship programs: a survey of the Society for Healthcare Epidemiology of America Research Network. Infect Control Hosp Epidemiol 2019;40:1074–1076. 31328703 10.1017/ice.2019.194 PMC7165360

